# Impact of parental lifestyle patterns in the preconception and pregnancy periods on childhood obesity

**DOI:** 10.3389/fnut.2023.1166981

**Published:** 2023-05-18

**Authors:** Marion Lecorguillé, Mireille C. Schipper, Aisling O’Donnell, Adrien M. Aubert, Muriel Tafflet, Malamine Gassama, Alexander Douglass, James R. Hébert, Blandine de Lauzon-Guillain, Cecily Kelleher, Marie-Aline Charles, Catherine M. Phillips, Romy Gaillard, Sandrine Lioret, Barbara Heude

**Affiliations:** ^1^Université Paris Cité and Université Sorbonne Paris Nord, Inserm, INRAE, Center for Research in Epidemiology and StatisticS (CRESS), Paris, France; ^2^The Generation R Study Group (Na 29-15), Erasmus University Medical Center, CA, Rotterdam, Netherlands; ^3^School of Public Health, Physiotherapy and Sports Science, University College Dublin, Dublin, Ireland; ^4^Ined, Inserm, EFS, Joint Unit Elfe, Aubervilliers, France; ^5^Cancer Prevention and Control Program and Department of Epidemiology and Biostatistics, Arnold School of Public Health, University of South Carolina, Columbia, SC, United States; ^6^Department of Nutrition, Connecting Health Innovations, LLC, Columbia, SC, United States

**Keywords:** 1,000 days, parental lifestyle patterns, preconception, childhood obesity, parental diet, parental physical activity, parental smoking, parental BMI

## Abstract

**Introduction:**

High prevalence of overweight and obesity already observed in preschool children suggests the involvement of early-life risk factors. Preconception period and pregnancy are crucial windows for the implementation of child obesity prevention interventions with parental lifestyle factors as relevant targets. So far, most studies have evaluated their role separately, with only a few having investigated their potential synergistic effect on childhood obesity. Our objective was to investigate parental lifestyle patterns in the preconception and pregnancy periods and their association with the risk of child overweight after 5 years.

**Materials and methods:**

We harmonized and interpreted results from four European mother-offspring cohorts participating in the EndObesity Consortium [EDEN, France; Elfe, France; Lifeways, Ireland; and Generation R, Netherlands] with data available for 1,900, 18,000, 1,100, and 9,500 families, respectively. Lifestyle factors were collected using questionnaires and included parental smoking, body mass index (BMI), gestational weight gain, diet, physical activity, and sedentary behavior. We applied principal component analyses to identify parental lifestyle patterns in preconception and pregnancy. Their association with risk of overweight (including obesity; OW-OB) and BMI *z*-scores between 5 and 12 years were assessed using cohort-specific multivariable logistic and linear and regression models (adjusted for potential confounders including parental age, education level, employment status, geographic origin, parity, and household income).

**Results:**

Among the various lifestyle patterns derived in all cohorts, the two explaining the most variance were characterized by (1) “high parental smoking, low maternal diet quality (and high maternal sedentary behavior in some cohorts)” and, (2) “high parental BMI and low gestational weight gain.” Patterns characterized by high parental BMI, smoking, low diet quality or high sedentary lifestyle before or during pregnancy were associated with higher risk of OW-OB in children, and BMI *z*-score at any age, with consistent strengths of associations in the main cohorts, except for lifeways.

**Conclusion:**

This project provides insight into how combined parental lifestyle factors in the preconception and pregnancy periods are associated with the future risk of child obesity. These findings are valuable to inform family-based and multi-behavioural child obesity prevention strategies in early life.

## 1. Introduction

Childhood obesity is a major public health issue associated with short and long-term adverse consequences, such as psychological problems and lower educational attainment, and a higher risk for many harmful comorbidities later in life, such as type 2 diabetes mellitus, dyslipidemia, and coronary heart disease (1). Around 40 million children under 5 years are living with overweight or obesity worldwide (2). High prevalence of obesity in preschool children suggests the involvement of early-life risk factors. However, the multiple causes of childhood obesity are not fully understood. The first 1,000 days, from conception to two years of age, represent a unique window of opportunity to implement child obesity prevention strategies and increase future health benefits (2).

Several systematic reviews have synthesized the results of observational studies linking suboptimal maternal lifestyle factors in preconception and during pregnancy with the risk of childhood obesity (3–5). Recent evidence showed that the preconception period is a key determinant of pregnancy success and next generation health (6, 7). Such maternal lifestyle factors in the preconception period can directly influence oocyte quality, embryonic, and placental development in the very first weeks of pregnancy, thus predisposing to a higher risk of childhood obesity and its related comorbidities (8). During pregnancy, unfavorable maternal lifestyle and health factors could also lead to abnormal fetal development and predispose to childhood obesity (3, 5). Maternal overweight, obesity, excessive gestational weight gain (GWG), unhealthy diet, smoking, and sedentary behaviours are the most prevalent adverse lifestyle factors before and during pregnancy (3, 5, 7). A recent meta-analysis, including more than 162,000 mother–children pairs from 37 cohort studies in high-income countries, reported that maternal overweight at conception and excessive GWG were independently associated with the risk of overweight and obesity during childhood and adolescence (9). Other associations between low maternal diet quality during pregnancy and overweight or obesity in children have been reported; however, results are inconsistent (10, 11).

So far, most studies have evaluated the effects of early-life maternal factors separately, with only a few having used patterning approaches to investigate their combined and potentially synergistic effect on childhood obesity. Furthermore, paternal lifestyle factors during the first 1,000 days have scarcely been considered, despite their likely influence on both the mother’s and the child’s lifestyle. Clustering of parental risk factors is a major public health concern, because they not only have individual effects on childhood obesity risk, but also interact with each other, which can further increase the risk of childhood obesity. There has been a consensus that family-based interventions, targeting the two generations, i.e., parents and their children, tend to be more effective (12). Lastly, strategies to prevent childhood obesity should start as early as possible, before birth and even in the preconception period; based on the scientific evidence of the life course epidemiology, developmental programming around the time of conception, and parental predisposition to adopt healthier behaviors for the future child (7).

In this context, our aim was to identify parental-based lifestyle patterns in the preconception and pregnancy periods and to assess their association with childhood overweight and obesity between 5 and 12 years. To this end, we have performed cohort-specific analyses in four European cohorts implementing a harmonized data and analysis approach.

## 2. Methods

### 2.1. Study population

This project involves four mother—offspring cohort studies across 3 European countries within the EndObesity consortium, which was established in 2020 with an overarching aim to develop, implement and evaluate innovative, multi-disciplinary strategies for prevention of childhood overweight by targeting family-based lifestyle factors in the 1,000 days. These cohorts/longitudinal follow-ups include the study on the pre- and early postnatal determinants of child health and development (EDEN; recruitment of ≈1,900 pregnant women from 27 January 2003 to 6 March 2006); the French national birth cohort (Elfe; recruitment of ≈18,000 children in 4 waves in 2011) in France; the Lifeways Cross-Generation Cohort Study (Lifeways; recruitment of ≈1,100 participants from 2 October 2001 to 4 April 2003) in Ireland; and The Generation R Study (Generation R; recruitment of ≈9,500 participants pregnant women with an expected delivery date between 1 April 2002 and 31 January 2006) in the Netherlands.

Briefly, the EDEN mother–child study is a prospective cohort that was designed to evaluate the early, pre-, and post-natal determinants of child health and development. Study participation was proposed to all women visiting the prenatal clinic before 24 weeks’ gestation. Between 2003 and 2006, 2002 (53%) pregnant women 18 to 45 years old were recruited in two centers: Nancy and Poitiers hospitals (13) and 89.2% of fathers agreed to participate. Children have been followed-up for up to 12 years, by visits to research centres and questionnaires mailed to parents. The ELFE study is a French national longitudinal birth cohort with more than 18,000 children included at birth (14). Recruitment took place on 25 selected days during four periods in 2011. Participation in the cohort was proposed to women who gave birth in 349 maternity hospitals randomly selected among the 544 public and private maternity hospitals in metropolitan France. Among eligible mothers, 51% agreed to participate (*N* = 18,040), and a sub-sample of 5,600 fathers have also participated. Children will be followed-up until 20 years of age by questionnaires mailed to parents or telephone interviews and visit to doctors at 2, 4 and 12 yrs.

The Lifeways Cross-Generation Cohort Study is a prospective family study which aimed to document health status, diet, and lifestyle in the family members and establish patterns and links across generations (15). Mothers (*n* = 1,124) were initially recruited by a midwife during their first antenatal visit in two maternity hospitals in the Republic of Ireland between 2001 and 2003. The participating mothers’ partners (biological father) were also directly contacted by the Lifeways research team at recruitment (participant mothers having given their contact details at their booking visit) and invited to participate (*n* = 333 agreed). Longitudinal follow-up was conducted with linkage data to hospital and general practice records and examination of children when aged on average 5 and 9 years.

Generation R Study is a population based prospective cohort study from early pregnancy onwards in Rotterdam, the Netherlands (16). Among participants, 9,978 mothers were enrolled in the study, of whom 91% (*n* = 8,800) were between early pregnancy and until birth. Maternal data has been collected repeatedly during pregnancy by physical examinations, biological samples and questionnaires. Fathers were invited to participate and 71% (*n* = 6,347) agreed. The fathers were assessed once during the first prenatal visit to the research center using physical examinations and questionnaires. At the ages of 5 and 9 years all children were invited to the research center for detailed cardiometabolic data collection by physical examination, biological samples and questionnaires. Repeated measurements of child growth from birth until the age of 13 were used for the calculations of adiposity peak and rebound. The study design for each cohort has been described in detail elsewhere (13–16). We retained one out of each twin pair in the Elfe and Lifeways cohorts involving twin births on a random basis. Twins in Generation R were excluded from analyses (*n* = 106) because the percentage of missing data was higher in this group and imputation was questionable due to the inherent specificities of this population. The characteristics of each study and numbers of participants included for the current analysis are summarized in Table 1.

**Table 1:** Characteristics of the cohort populations.

	EDEN* (*N* = 1981)	Elfe* (*N* = 17,904)	Generation R* (*N* = 8,765)	Lifeways* (*N* = 932)
Maternal characteristics
Age (years)	29.5 ± 4.9	30.2 ± 5.1	30.3 ± 5.3	29.6 ± 5.9
Born abroad	4.1 (79)	13.7 (2327)	34.3 (2897)	NA**
Low	7.5 (144)	8.5 (1524)	11.0 (901)	18.8 (171)
Medium	39 (745)	34.9 (6240)	46.0 (3753)	31.3 (285)
High	53.5 (1021)	56.6 (10136)	43.0 (3508)	49.9 (454)
Employed/self-employed	76.3 (1461)	78.8 (14016)	72.9 (4772)	67.2 (620)
Primiparous	44.6 (848)	45.9 (8110)	56.0 (4810)	45.9 (421)
Pre-pregnancy smoking (Yes)	36.1 (688)	41.9 (7392)	39.1 (2822)	52.3 (404)
Underweight	8.6 (162)	7.9 (1385)	4.4 (320)	2.9 (22)
Normal	65.1 (1226)	64.9 (11431)	67.0 (4826)	69.4 (532)
Overweight	17.6 (332)	17.2 (3038)	19.4 (1399)	19.6 (150)
Obese	8.7 (164)	10 (1764)	9.1 (658)	8.2 (63)
Pre-pregnancy E-DII	0.5 ± 1.7	NA	NA	NA
Pre-pregnancy DASH	24 ± 4.3	NA	NA	NA
Maternal GWG (kg)	13.4 ± 4.8	13.2 ± 5.5	10.3 ± 5.0	NA
Pregnancy smoking	27.1 (520)	19.9 (3514)	18.0 (1290)	21.6 (192)
Pregnancy E-DII	0.92 ± 1.7	NA	−0.3 ± 1.1	0.43 ± 1.8
Pregnancy DASH	23.9 ± 4.3	24.0 ± 4.4	24.0 ± 4.6	23.7 ± 4.6
Paternal characteristics
Age (years)	32.00 ± 5.9	33.3 ± 6.2	33.4 ± 6.0	32.1 ± 6.2
Born abroad	7.3 (138)	15.1 (2475)	35.6 (2785)	NA**
Low	10 (191)	8.7 (1184)	8.2 (423)	31.8 (261)
Medium	46.3 (885)	40.3 (5513)	41.2 (2115)	26.9 (221)
High	43.7 (835)	51 (6966)	50.6 (2602)	41.3 (339)
Employed/self-employed	91.1 (1709)	90.1 (15769)	91.3 (4356)	99.1 (751)
Pre-pregnancy smoking (Yes)	43.9 (740)	NA	NA	52.2 (129)
Underweight	1.1 (19)	0.8 (94)	1.0 (59)	1.1 (3)
Normal	54.2 (966)	55 (6855)	49.3 (3046)	33.3 (89)
Overweight	36.2 (645)	36.1 (4500)	41.0 (2534)	49.8 (133)
Obese	8.6 (153)	8.1 (1004)	8.7 (539)	15.7 (42)
Pregnancy smoking	40.2 (697)	35.2 (4407)	44.8 (3282)	32.5 (89)
Pregnancy E-DII	NA	NA	NA	1.5 ± 1.7
Household income
1st quartile (lowest)	16.7 (318)	14 (2145)	20.1 (1306)^a^	62.9 (531)^d^
2nd quartile	29.7 (564)	21 (3207)	25.0 (1621)^b^	37.1 (313)^e^
3rd quartile	26.3 (500)	33 (5047)	54.9 (3563)^c^	NA
4th quartile (highest)	27.2 (517)	31.9 (4875)	NA	NA

Values are means ± SD for continuous variables and % (*n*) for categorical variables. BMI, body mass index; DASH, dietary approach to stop hypertension; E-DII, energy-adjusted dietary inflammatory index; GWG, gestational weight gain. *Data characteristics based on the selection of the population before imputation of the lifestyle factors: EDEN *N* = 1981 (with at least one factor in the maternal model in preconception); Elfe *N* = 17,904 (with at least one factor in the family model in pregnancy); Gen-R *N* = 8,765 (with at least one factor in the parental pregnancy model), Lifeways *N* = 932 (with at least one factor in the parental pregnancy model). **Maternal birth outside Ireland was an exclusion criterion in Lifeways.

a Value for category (<1,200 or 1,200 euro).

b Value for category (1200–2,200 euro).

c Value for category (>2,200 euro).

d <600£/week.

e ≥600£/week.

### 2.2. Ethics statement

All studies have been approved by the local ethics review committees and written informed consents were obtained from all families (Supplementary Table S1).

### 2.3. Candidate variables for parental-based lifestyle

We selected candidates for parental-based lifestyle factors in preconception and pregnancy possibly associated with childhood overweight from an extensive literature search and priori hypotheses. The literature search included observational and interventional studies, as well as systematic reviews and meta-analyses (3–5). We did a first work to organize the different factors identified from the literature search, according to the period of interest and following the level of evidence, from the lowest possible early markers to the highest probable early markers of obesity in preconception and during pregnancy. Our classification was based on how different reviews (3–5) have graded the level of evidence according to the number of studies reporting an association between the factor and risk of obesity in children. Then, we compared this list to the available information in the EndObesity cohorts. We conducted a data inventory to identify available variables within each cohort. Preference was given to the use of variables harmonized between the four cohorts from previous work conducted as part of either the H2020 LifeCycle (17) or the ERA-Net HDHL APLHABET (18) projects. The LifeCycle project is a Horizon 2020-funded international project, which has developed a harmonized set of variables (including those describing lifestyle) in a Europe-wide network of cohort studies started in early life. Protocols for LifeCycle harmonization are available in free access (19). The ALPHABET project is a European consortium composed of seven longitudinal birth cohort studies, the food frequency questionnaires of which were harmonized, then DASH and DII scores derived following a common methodology. All the harmonized variables for parental-based lifestyle used in our project are described in Supplementary Table S2 and Supplementary Text 1.

In preconception, we retained the following factors: maternal pre-pregnancy and paternal body mass index [BMI = weight (kg)/height(m^2^)], smoking before pregnancy (No, <10 cig/day defined as low smoking, ≥10 cig/day defined as high smoking), and dietary quality and inflammatory potential (Supplementary Table S2). We selected BMI as a marker of an obesogenic lifestyle by considering that it was related to other lifestyle factors. During pregnancy, we used similar information including maternal pre-pregnancy BMI, paternal BMI, smoking during pregnancy (No, <10 cig/day, ≥10 cig/day), diet, and additionally included maternal GWG (calculated as measured weight at the end of pregnancy (third trimester in Generation R) minus weight at conception as reported by mothers) and information on physical activity level when available (Supplementary Table S2). If pre-pregnancy weight was not available, we used early pregnancy weight closest to conception, limited to 1st trimester (<12 weeks). Data were collected using self-administered health and lifestyle questionnaires at inclusion or at birth, face-to-face interviews or information collected in medical records.

### 2.4. Dietary scores

The usual diet of women was assessed using validated semi-quantitative food frequency questionnaires (FFQs) completed during pregnancy or at birth. The FFQs were based on different periods: the maternal diet during the year before pregnancy in EDEN, at early trimester (12–16 weeks of gestation) in Lifeways, over the three preceding months in Generation R (thereby covering dietary intake in the first trimester of pregnancy), or for the last three months of pregnancy in EDEN and Elfe. FFQs comprised a variety of items in each cohort, along with their frequencies of consumption and portion sizes, thus allowing us to assess the daily intake of each item (g/d). The micronutrient, macronutrient and total energy intake were also calculated at the individual level. Description of the methodology to assess the dietary data was published in detail elsewhere (10, 20–23). A sub-sample of 998 fathers completed a short FFQ based on diet in the months before the beginning of their partner’s pregnancy in the Elfe study (23), while in Lifeways, 333 fathers also completed a similar FFQ providing information on their habitual diet for the last year from after booking visit when mothers were recruited (10).

Diet quality was assessed by degree of adherence to the dietary approach to stop hypertension (DASH) score, while the dietary inflammatory potential was determined using the energy-adjusted dietary inflammatory index (E-DII) (18). We collectively decided to use established *a priori* scores such as the DASH (18) and the E-DII (24) rather than create *a posteriori (data-driven)* dietary patterns that would possibly be cohort-dependent. In brief, the DASH score measured the consumption of eight food components based on cohort-quintile rankings for an overall score ranking from 8 to 40 points with a higher score characterizing a higher diet quality (18). The E-DII score measured the intake of food parameters with a pro- or anti-inflammatory potential. All the food parameter-specific E-DII scores are summed to obtain the overall E-DII score with a positive score indicating a more proinflammatory diet, whereas a negative score indicating a more anti-inflammatory diet (24). For EDEN, Lifeways and Generation R, these two scores were for maternal diet generated using a harmonised procedure (described in Supplementary Text 1) within the ERA-Net HDHL ALPHABET project (18, 25). In Elfe, the maternal DASH score was created following a similar methodology among mothers, but was not available for fathers; we thus used the food groups that were coherent with those constituting the DASH score (such as fruit, vegetables, meat), and for which we had at least 50% of consumers. For paternal diet, E-DII was available in Lifeways only.

### 2.5. Physical activity data

Physical activity data were available in the EDEN, Elfe and Lifeways cohorts. In EDEN, women completed an adapted French version of the Baecke questionnaire, validated measure of habitual physical activity for adults (26, 27). This questionnaire was administered at the 24–28 weeks pregnancy visit and refers to the frequency of physical activities during the first trimester of pregnancy. In Elfe, 15,544 women completed the self-administered Pregnancy Physical Activity Questionnaire, specifically designed and validated to assess physical activities among pregnant women (28, 29). Respondents were asked to report the time spent participating in 32 activities during the last three months of pregnancy. The different questionnaires reported information on occupational, sports, leisure-time activity, household/caregiving and sedentary activities (see Supplementary Text 1). In Lifeways, at recruitment during their first antenatal visit mothers were given a questionnaire with sections relating to general health, lifestyle and social characteristics to complete and return by email. The mothers’ participating partner (biological father) also returned the baseline self-completed questionnaire. The questions relating to physical activity have been used in the Irish National Surveys of Lifestyle Attitudes and Nutrition 1998, 2002, 2007 (30–32). Respondents were asked “considering a 7 day period (a week), how many times on average do you do the following kinds of exercise (mild, moderate and strenuous, with examples provided for each) for more than 20 min during your free/leisure time?”

### 2.6. Outcomes

Child BMI was assessed using weight and height measurements collected from the clinical examinations, the child’s health booklet or assessed by parents between 5 and 12 years. We considered two or three age ranges depending on each cohort, which corresponded either to the clinical examinations in the study or to key points in the child’s development. In each cohort, we used the International Obesity Task Force (IOTF) references and cut-offs to assess BMI *z*-score and risk of overweight, obesity and thinness, taking child sex and age into account (33). We investigated the risk of child overweight and obesity (OW-OB) by combining the categories of overweight/obesity vs. thin/normal BMI (the equivalent of <25 kg/m^2^).

We considered as secondary outcome the age at adiposity rebound AR (available in EDEN, Elfe and Generation R); the timing of AR, independent of BMI itself, being a relevant predictor of obesity in later childhood and adulthood (34).

### 2.7. Covariates

Important covariates were identified and harmonized for subsequent analyses. These include maternal and paternal age (in years), education level according to the International Standard Classification of Education (low [no education; early childhood; pre-primary; primary; lower secondary or second stage of basic education]/medium [Upper secondary, Post-secondary non-tertiary]/high [Short cycle tertiary, Bachelor, Masters, Doctoral or equivalent]), employment status ((self)employed/not employed: unemployed, housewife, student, inactive/other); geographical origin (born outside the cohort country or not); maternal parity (primiparous/multiparous); and total yearly household income categorized into quartiles in EDEN and Elfe, based on national distribution of household yearly income (low/medium-low/medium-high/high). The household’s total net income per week (take-home family weekly income from all sources including social benefits) was categorized as <600 or ≥600 £/week in Lifeways. In the Generation R cohort, household income data were categorized as follows: ≤1,200, 1,201–2,200, >2,200 €/month. These data were originally collected by questionnaires (interviewer- or self-administered) or abstracted from birth medical records. Data are expressed in different units and categories in different studies, thus we used harmonized data for downstream analysis.

### 2.8. Statistical analyses

#### 2.8.1. Principal component analysis

We derived “lifestyle patterns” using principal component analysis (PCA) (35). PCA is both a global and an integrated method to synthetize the information contained in a large number of correlated factors into a smaller number of independent dimensions (or “components”). Their number was selected considering eigenvalues >1.0, the scree plot, and their interpretability. To interpret the results and provide a label for a given pattern, we considered the variables most strongly related to that pattern, and those for which the absolute value of the factor loading (which is the correlation of each standardized variable with the given lifestyle pattern) was >0.30. An individual score for each lifestyle pattern was calculated by summing the observed standardized values of each variable weighted according to the PCA factor loadings. Because PCA is sensitive to outliers, we also checked results by excluding some extreme values, when necessary (i.e., for maternal BMI). Analyses were conducted within each cohort separately with the maximum of available variables per cohort: we considered that at least 4 variables were requested for a given period (preconception and pregnancy) to compute the PCAs. Due to insufficient available information related to preconception in the Lifeways cohort, we did not consider this cohort for this period. PCAs were performed using imputation with the missMDA R package (36). This package can be used to perform single imputation to complete data involving continuous, categorical and mixed variables (i.e., data with different types of variables). As it is based on a principal component method, imputation considers both similarities between individuals and correlations between variables. We used the regularized iterative PCA algorithm for imputation of the missing values using the values predicted by iterative PCA until convergence. When using imputation, we selected individuals with information on at least one variable to be included in the PCA. PCAs were run at each period separately and in two steps: first including maternal variables only and, second, using both maternal and paternal variables.

### 2.9. Regression models

We assessed the associations of the identified maternal or parental lifestyle patterns at each period, with the risk of child OW-OB between 5 and 12 years of age using multivariable logistic regression models. Based on literature and identification of available information in the different studies, the following set of *a priori* covariates was adjusted for: maternal and paternal age, education level, employment status, geographic origin, family household income and maternal parity. Secondly, we studied the associations between the identified lifestyle patterns, included in the same model, with both the child’s BMI *z*-score and age at AR (when available) using multivariable linear regressions (adjusted for the same confounders desribed above, and for child sex when the outcome is age at AR). We checked that there was no collinearity between all covariates by using the variance inflation factor (considering the threshold of collinearity when variance inflation factor, VIF > 3). Confounders were imputed using the “MICE” R package that imputes incomplete multivariate data by chained equations. We generated 20 imputed datasets, using logistic regression, multinomial logit model, and predictive mean matching for categorical and quantitative variables (37) (see Supplementary Tables S3–S6). Finally, as a sensitivity analysis, we re-ran all the analyses restricted to complete cases, and results were consistent (data not shown). Specific estimates were obtained for each cohort. Analyses were performed using R studio (for PCA and imputation), and SAS or R studio (for regression models). Statistical significance was defined as *p*-values <0.05, two-tailed.

## 3. Results

### 3.1. Descriptive results

Parental characteristics of the samples included in the analyses are presented in Table 1. On average, across cohorts, mothers, and fathers were 30 and 33 years old. The prevalence of parental obesity varied between 8.2 and 10% for mothers; and 8.1 and 15.7% for fathers. From 50 to 57% of mothers had a high education level in the French cohorts and Lifeways, whereas it was 43% in Generation R. Overall, 36 to 42% of women smoked before pregnancy in EDEN and Elfe, 39% in Generation R and 52% in Lifeways. This percentage decreases during pregnancy to 27% in EDEN, 20% in Elfe, 18% in Generation R and 22% in Lifeways. The percentage of paternal smoking before pregnancy was 44% in EDEN and 52% in Lifeways.

### 3.2. Preconception parental lifestyle patterns and associations with child risk of overweight and BMI *z*-score

We identified various maternal and parental lifestyle patterns in preconception (Table 2). Consistent patterns were identified in EDEN and Generation R, the first one being characterized by “parental smoking and low maternal diet quality.” As expected, the DASH and the E-DII scores were associated with opposite factor loadings to this pattern since they are negatively correlated. Low diet quality was used to label patterns characterized by high DASH and low E-DII scores. The second pattern was labelled “high parental BMI and low smoking.” A third pattern was characterized by “high parental smoking, BMI, and high maternal diet quality.” Consistent patterns were identified when only maternal factors were included in the PCAs (Table 2). In Elfe, three other patterns were characterized by “low paternal diet quality,” “high parental BMI, maternal smoking and low paternal consumption of vegetables,” “high paternal BMI, paternal consumption of vegetables and low maternal smoking.” Factor loadings of the parental lifestyle patterns in the preconception period are presented in Supplementary Table S7. We reported that the parental lifestyle pattern characterized by a high parental BMI and low smoking was positively associated with child risk of OW-OB at 5 years in both EDEN and Generation R (OR [95% CI] = 1.59 [1.33;1.89], and 1.63 [1.52;1.74] respectively, per 1 standard-deviation (SD) increase in the score) (Table 3). Consistent findings were observed at later ages (until 12 years) regarding risk of OW-OB, higher child BMI *z*-score (Supplementary Table S8), and with a similar maternal lifestyle pattern (Supplementary Table S9). The pattern characterized by high parental smoking, BMI and high maternal diet quality was also associated with higher risk of OW-OB at 5 years in EDEN and Generation R (OR = 1.37 [1.11;170], and 1.34 [1.25;1.44] respectively, per 1 SD increase), and higher child BMI *z*-score (*p*-values < 0.05). In Elfe, the pattern with high parental BMI, maternal smoking, and low paternal consumption of vegetables was positively associated with the risk of OW-OB (OR = 1.34 [1.01; 1.79]). Finally, an increase of score in the pattern characterized by high parental smoking, and low maternal diet quality was associated with higher risk of OW-OB at 5 and 9 years in Generation R (OR = 1.13 [1.07; 1.19] at 5 years) (Table 3).

**Table 2:** Summary of PCA results in preconception and pregnancy.

	EDEN	Elfe^a^	Gen R	Lifeways
Preconception period				
Family patterns in preconception	*N* = 1981 Parental smoking and low maternal diet quality (32.9%) High parental BMI and low smoking (20.4%) High parental smoking, BMI, and high maternal diet quality (17.8%)	*N* = 917 Low paternal diet quality (21.1%) High parental BMI, maternal smoking and low paternal consumption of vegetables (18%) High paternal BMI, consumption of vegetables and low maternal smoking (15.1%)	*N* = 8,352 High parental smoking and low maternal diet quality (31.8%) High parental BMI and low smoking (19.7%) High parental smoking, high paternal BMI and high maternal diet quality (16.9%)	NA
Maternal lifestyle patterns in pre-conception	*N* = 1981 High smoking and diet inflammatory potential & low DASH (43.8%) High BMI and rather low smoking (25.3%)	NA	*N* = 8,156 High smoking, diet inflammatory potential & low DASH (42.1%) High BMI, and low smoking (25.6%)	NA
Pregnancy period				
Family patterns in pregnancy	*N* = 1962 High parental smoking, low maternal diet quality and low leisure PA (20.2%) Low parental BMI and high GWG (13.6%) Parental smoking, low GWG and low maternal work PA (11.3%)	*N* = 17,904 High parental smoking, low maternal diet quality and maternal sedentary (16.3%) Low parental BMI and high GWG (13.4%) High maternal physical activity (12.8%) High GWG, maternal sedentary and occupational PA (10.6%)	*N* = 8,765 High parental smoking, low maternal diet quality (27.2%) Low parental BMI, high GWG (18.9%)	*N* = 932 High parental smoking, inflammatory diet, low maternal DASH, and rather low paternal PA (25.2%)
Maternal lifestyle patterns in pregnancy	*N* = 1925 Low smoking, high diet quality and leisure PA (23.5%) Low BMI and high GWG (15.9%) Smoking and high sport PA (13.1%)	*N* = 17,879 High BMI, smoking, low diet quality, high household PA and sedentary (17.4%) High diet quality, and high PA (15.9%) Low BMI, high GWG, smoking, high PA (15.6%)	*N* = 8,546 High BMI, smoking, low diet quality (34.7%) Low BMI and high GWG, and smoking (24.3%)	*N* = 931 Smoking, and low diet quality (35.4%) Low BMI and high PA (21.1%)

Lifestyle pattern labels and proportion of variance explained (%). PA, physical activity. Results are presented with imputation for all cohorts.

a Elfe results on complete cases for the preconception period. Contrary low diet quality = low DASH and high E-DII scores.

**Table 3:** Associations of preconception family lifestyle patterns with child risk of overweight between 5 and 12 years.

IOTF overweight/obesity adjusted OR [95% CI]										
EDEN				Elfe				Gen R		
	**5.5 years (*N* = 1,143)**	**8 years (*N* = 737)**	**12 years (*N* = 706)**		**5 years (*N* = 638)**	**7 years (*N* = 493)**	**9 years (*N* = 352)**		**5 years (*N* = 5,778)**	**9 years (*N* = 4,926)**
“Parental smoking and low maternal diet quality”	1.12 [0.94–1.35]	0.90 [0.74–1.09]	1.21 [1.00–1.47]*	“Low paternal diet quality”	1.06 [0.77–1.46]	1.10 [0.78–1.56]	0.82 [0.53–1.27]	“High parental smoking, and low maternal diet quality”	1.13 [1.07–1.19]*	1.17 [1.10–1.24]*
“High parental BMI and low smoking”	1.59 [1.33–1.89]*	1.64 [1.35–2.01]*	1.57 [1.28–1.92]*	“High parental BMI, maternal smoking and low paternal consumption of vegetables”	1.34 [1.01–1.79]*	1.84 [1.34–2.53]*	1.88 [1.28–2.76]*	“High parental BMI and low smoking”	1.63 [1.52–1.74]*	1.69 [1.57–1.82]*
“High parental smoking, BMI and high maternal diet quality”	1.37 [1.11–1.70]*	1.54 [1.21–1.97]*	1.44 [1.12–1.85]*	“High paternal BMI, high consumption of vegetables and low maternal smoking”	1.78 [1.31–2.44]*	1.37 [0.99–1.90]	1.51 [1.04–2.19]*	“High parental smoking, high paternal BMI, and high maternal diet quality”	1.34 [1.25–1.44]*	1.36 [1.26–1.47]*

Lifeways study is not included because of the lack of information on behaviors during the pre-conception period. Models are adjusted for parental age, born abroad or not, parental education, parental employment status, parity, household income. Results are presented using imputed lifestyle patterns (except for Elfe). *Significant associations *p* < 0.05. **IOTF overweight/obesity vs. underweight/normal BMI (reference). OR values of logistic regression and 95% CIs for 1 SD increase in lifestyle pattern score.

### 3.3. Pregnancy parental lifestyle patterns and associations with child risk of overweight and BMI *z*-score

Regarding the pregnancy period (Table 2), a first pattern was consistent with the one described in preconception with “parental smoking and low maternal diet quality” as common characteristics between all cohorts. A second one was essentially defined by weight status as “low parental BMI and high GWG” in EDEN, Elfe and Generation R. Of note, a great coherence was observed when only maternal factors were included (Table 2).

In generation R, we observed a positive association between the lifestyle pattern with “high parental smoking, low maternal diet quality” and the risk of OW-OB, while the parental lifestyle pattern characterized by a low parental BMI and high GWG was negatively associated with risk of OW-OB and child BMI *z*-score in EDEN, Elfe and Generation R (Table 4 and Supplementary Table S10). We also found that another pattern characterized by parental smoking, low maternal diet quality and maternal sedentary behaviour was positively associated with a higher risk of OW-OB in Elfe (OR [95% CI] = 1.31 [1.23; 1.40]), and higher child BMI *z*-score at 5 years. In Elfe, the one SD score increase on the pattern labeled “high GWG, sedentary and occupational PA” was also associated with higher risk of OW-OB (OR = 1.17 [1.09; 1.26]), and higher child BMI *z*-score. Associations were consistent at later ages. No significant associations were reported in Lifeways and effect sizes were smaller than in the other cohorts. Consistent findings were observed with maternal lifestyle patterns (Supplementary Table S11).

**Table 4:** Associations of pregnancy family lifestyle patterns with child risk of overweight between 5 and 12 years.

IOTF overweight/obesity adjusted OR [95% CI]**													
EDEN				Elfe			Gen R			Lifeways			
	**5.5 years (*N* = 1,143)**	**8 years (*N* = 737)**	**12 years (*N* = 706)**		**5 years (*N* = 9,335)**	**7 years (*N* = 3,826)**	**9 years (*N* = 3,339)**		**5 years (*N* = 6,117)**	**9 years (*N* = 5,227)**		**5 years (*N* = 534)**	**9 years (*N* = 289)**
“High parental smoking, low maternal diet quality and low leisure PA”	1.20 [1.00–1.44]	1.11 [0.90–1.36]	1.14 [0.95–1.39]	“High parental smoking, low maternal diet quality and maternal sedentary”	1.31 [1.23–1.40]*	1.34 [1.21–1.48]*	1.32 [1.19–1.47]*	“High parental smoking, low maternal diet quality”	1.12 [1.06–1.18]*	1.14 [1.08–1.21]*	“High maternal and paternal smoking & inflammatory diet, low maternal DASH diet, low paternal PA”	1.06 [0.92–1.23]	1.04 [0.85–1.27]
“Low parental BMI and high GWG”	0.70 [0.58–0.83]*	0.67 [0.54–0.81]*	0.76 [0.62–0.93]*	“Low parental BMI and high GWG”	0.83 [0.78–0.88]*	0.71 [0.65–0.78]*	0.73 [0.66–0.80]*	“Low parental BMI, high GWG”	0.75 [0.71–0.79]*	0.71 [0.67–0.76]*			
“Parental smoking, low GWG and low maternal work PA”	1.01 [0.82–1.26]	0.80 [0.62–1.01]	1.14 [0.91–1.44]	“High maternal physical activity”	0.95 [0.89–1.02]	1.00 [0.90–1.10]	0.93 [0.83–1.04]						
				“High GWG, sedentary and occupational PA”	1.17 [1.09–1.26]*	1.25 [1.12–1.39]*	1.23 [1.10–1.38]*						

Models are adjusted for parental age, born abroad or not, parental education, parental employment status, parity, household income. For Lifeways, models were not adjusted for paternal employment status (almost all cases reported being employed/self-employed) and on country of birth (maternal birth outside Ireland was an exclusion criterion). *Significant associations *p* < 0.05. **IOTF overweight/obesity vs. underweight/normal BMI (reference). OR values of logistic regression and 95% CIs for 1 SD increase in lifestyle pattern score.

### 3.4. Associations with age at adiposity rebound (AR)

The associations of parental lifestyle patterns with age at AR are documented in Table 5. Results for the associations with maternal lifestyle patterns are shown in Supplementary Tables S9, S11.

**Table 5:** Associations of preconception and pregnancy family lifestyle patterns with age at adiposity rebound.

Age at adiposity rebound (days) adjusted *β* [95% CI]					
Preconception family patterns					
EDEN (*N* = 1,415)		Elfe (*N* = 649)		Gen R (*N* = 5,922)	
“Parental smoking and low maternal diet quality”	−8.6 [−29.0; 11.7]	“Low paternal diet quality”	4.9 [−28.6; 38.4]	“High parental smoking, and low maternal diet quality”	−20.8 [−30.1; −11.6]*
“High parental BMI and low smoking”	−82.5 [−106.1; −58.9]*	“High parental BMI, maternal smoking and low paternal consumption of vegetables”	−105.5 [−139.7; −71.3]*	“High parental BMI and low smoking”	−114.7 [−126.0; −103.4]*
“High parental smoking, BMI, and high maternal diet quality”	−53.2 [−78.2; −28.1]*	“High paternal BMI, high consumption of vegetables and low maternal smoking”	−54.4 [−86.9; −22.0]*	“High parental smoking, high paternal BMI and high maternal diet quality”	−69.9 [−81.7; −58.1]*
Pregnancy family patterns					
EDEN (*N* = 1,415)		Elfe (*N* = 7,767)		Gen R (*N* = 6,299)	
“High parental smoking, low maternal diet quality and low leisure PA”	−14.3 [−35.2; 6.7]	“High parental smoking, low maternal diet quality and maternal sedentary”	−41.3 [−49.4; −33.1]*	“High parental smoking, low maternal diet quality”	−19.7 [−29.1; −10.4]*
“Low parental BMI and high GWG”	65.8 [42.8; 88.9]*	“Low parental BMI and high GWG”	52.0 [43.5; 59.8]*	“Low parental BMI, high GWG”	64.0 [53.2; 74.8]*
“Parental smoking, low GWG and low maternal work PA”	−5.6 [−31.8; 20.7]	“High maternal physical activity”	9.6 [1.6; 17.7]*		
		“High GWG, sedentary and occupation PA”	−24.4 [−33.4; −15.5]*		

Models are adjusted for parental age, born abroad or not, parental education, parental employment status, parity, household income and child sex. Results are presented using imputed lifestyle patterns (excepted for Elfe for the preconception period). *Significant associations *p* < 0.05. *β* values of linear regression and 95% CIs for 1 SD increase in lifestyle pattern score.

In preconception, we found that a parental lifestyle pattern defined by high parental BMI and low smoking was associated with an earlier age at AR in EDEN and Generation R [*β* (95% CI) = −82.5 (−106.1; −58.9) in days, per 1 SD increase in the score] and [*β* = −114.7 (−126.0; −103.4)], respectively. Similar findings were found with the maternal lifestyle pattern also characterized by a high BMI and low smoking (Supplementary Table S9). We observed that a pattern with “high parental smoking, and low maternal diet quality” was associated with decreased age at AR in Generation R [*β* = −20.8 (−30.1; −11.6)]. Finally, patterns with “high parental smoking, BMI and high maternal diet quality” in EDEN and Generation R or “high parental BMI, high paternal consumption of vegetables and low maternal smoking” in Elfe were associated with an earlier age at AR (Table 5).

In pregnancy, a parental lifestyle pattern defined by low parental BMI and high GWG was associated with later age at AR in all cohorts. In Generation R, a pattern with high parental smoking, low maternal diet quality was also associated with earlier age at AR. In Elfe, a pattern combining high parental smoking, low maternal diet quality and maternal sedentary lifestyle, was associated with reduced age at AR [*β* (95% CI) = −41.3 (−49.4; −33.1)] (Table 5); inversely, a pattern with higher maternal physical activity was associated with later age at AR. Lastly, a pattern defined by high GWG, sedentary lifestyle and occupational PA in Elfe was associated with an earlier age at AR [*β* (95% CI) = −24.5 (−33.4; −15.5]. Maternal patterns characterized by factors such as high BMI, smoking, low diet quality, high household PA and sedentary lifestyle were associated with an earlier age at AR (Supplementary Table S11).

## 4. Discussion

In this large collaborative project with harmonized participant data across four European cohorts, we identified various parental lifestyle patterns in the preconception period and during pregnancy with great consistency between countries. The two patterns explaining the most variance in pregnancy were characterized by “high parental smoking, low maternal diet quality and high maternal sedentary behavior” and by “high parental BMI and low gestational weight gain.” Overall, we found that lifestyle patterns characterized by high parental BMI, smoking, low diet quality or high sedentary lifestyle were positively associated with risk of OW-OB and child BMI *z*-score in children aged between 5 and 12 years. Although parental BMI was included in most of the patterns associated with the risk of obesity in children, it is noteworthy that a pattern independent of BMI and characterized by high parental smoking and low maternal diet quality in preconception, was associated with higher OW-OB in both the Generation R and EDEN cohorts. In Elfe, two other patterns of multiple behaviors in pregnancy, i.e., “parental smoking, low maternal diet quality and high maternal sedentary behavior” and “high GWG, sedentary behavior and occupational physical activity” were also associated with higher risk of OW-OB and higher child BMI *z*-score. Finally, it is also interesting to note that the observation of early AR was higher with suboptimal parental lifestyle patterns. The results of our study, which is, to the best of our knowledge, the first to simultaneously analyze child OW-OB risk and age at AR in relation to parental lifestyle patterns in the preconception and pregnancy periods, represent a novel and significant contribution to the knowledge base regarding family-based and multi-behavioral child obesity prevention strategies from early life onwards.

### 4.1. Interpretation

Obesity is a multifactorial disease and its etiology includes complex interactions between several factors (38). On a population level, it is estimated that up to 50% of childhood OW-OB could be attributed to modifiable family-centered early-life risk factors (39, 40). Family lifestyle factors are often correlated and co-vary, and may contribute synergistically to obesity development (38). The lifestyle pattern approach used in our study allowed us to account for the potential covariation and correlations between lifestyle factors, thus providing more holistic and integrated information for public health prevention. Growing evidence suggests the potential for multiple-behavior interventions to have a greater impact on public health than single-behavior interventions (41). Additionally, success in changing one or more lifestyle behaviors of the mother may also increase confidence or self-efficacy to improve correlated lifestyle behaviors of the father, and vice versa (41).

Recent scientific evidence has highlighted the need to include fathers in lifestyle and health interventions spanning the first 1,000 days. Paternal factors, including BMI and smoking, have been associated with fertility, imprinting of genes during spermatogenesis, embryonic, and future child development (42). Partners play a role in supporting future mothers, thus contributing to successful behavioural changes at the family level (43, 44). One study reported that the smoking status of a pregnant smoker’s partner influences her chances of quitting (44, 45). Other results showed that fathers may influence family food practices, and what is consumed within families is the product of interactions and negotiations between family members (46). Pregnancy is also an important time when parents, who play the role of health promoters, role models and educators in their offspring life, may be more susceptible, to make better lifestyle choices to positively impact their future baby’s health and influence their future behaviors (47). Parents are an integral target of interventions, given their influence in supporting and managing their children’s energy balance-related behaviors (43, 48).

Most studies have evaluated the effects of parental factors separately, with only few having studied the combined effect of these factors in the first 1,000 days (12, 49, 50). The Millennium Cohort Study investigated the dynamic relation between underlying family lifestyle (especially parental weight and smoking status, exclusive breastfeeding duration; and child behaviors between birth and 7 years) and childhood obesity until 7 years. They found that the proportion of variance in childhood weight status explained by underlying family lifestyle increases from 7.0% at age 3 to 11.3% by the age of 7 years, suggesting that improvements to the family lifestyle could substantially reduce the risk of childhood obesity (12). Another recent study of 114 mother–father–child triads evaluated the independent contributions of body composition, physical activity and sedentary time of both parents in pregnancy on growth trajectories of their offspring until 1 year of age (49). They reported a positive association between maternal GWG and paternal sedentary time and weight trajectories in girls, while there was a negative association with maternal moderate-to-vigorous PA (49). These results are in line with our observations regarding the association between lifestyle patterns characterized by high parental BMI or sedentary behaviors from preconception onwards and the risk of childhood obesity.

Regarding parental BMI, it is difficult to disentangle the relative contribution of environmental and genetic factors in the observed associations with offspring BMI or obesity risk. A systematic review found BMI heritability estimates from twin studies that ranged from 0.47 to 0.90 (51). Using a Mendelian randomisation approach, another recent study reported a low causal intrauterine effect of greater maternal BMI on later offspring adiposity and highlighted the importance of genetic transmission (52). Therefore, genetic inheritance could have a preponderant role in our results. However, recent evidence reports that the obesity epidemic cannot be solely explained by sudden changes in our genetic background, but is also attributed to the environment, energy balance-related behaviors or modifiable factors involved in programming including family lifestyle in the first 1,000 days (53, 54). In this context, parental lifestyle factors, including diet, physical activity or smoking are relevant behaviors to target. Moreover, the more lifestyle factors adopted during one pregnancy are optimal, the healthier are behaviors in subsequent pregnancies.

### 4.2. Strengths and limitations

To our knowledge, this is the largest multi-country study to evaluate the overall parental lifestyle patterns in preconception and during pregnancy to date. We aimed to reduce extraneous heterogeneity by harmonizing data across included studies, using information on several parental lifestyle factors in preconception and pregnancy: weight, smoking status, diet, and physical activity. We acknowledge that this harmonized approach can lead to some oversimplification in definitions and a subsequent loss of information for certain variables; and that more precise analyses could be carried out within each cohort using the initial non-harmonized variables. Besides, some information was missing, such as physical activity in Generation R, or was insufficient in the preconception period as in Lifeways. Nevertheless, this harmonized approach allowed us to report consistent findings across cohorts, in a greater number of children, thus reinforcing robustness of our conclusions. The use of multiple imputation techniques limited selection bias due to missing data in the lifestyle variables and confounders.

However, our findings should be interpreted with caution. Diet measures were based on FFQs with different degree of detail (number of food items, response categories, etc.) which could result in discrepancies in estimated intakes and diet scores. The fact that we have differences in the measurement of some lifestyle factors could explain why they are less frequently reported in the associations, and that BMI, which is more accurately measured, is an important factor in the patterns. Additionally, self-reported information on diet and physical activities are memory based and subject to well-known reporting and social desirability biases, that can introduce random error and information bias. However, FFQ is a validated tool, which limits such potential bias. Notwithstanding the fact that we performed our analyses using data from three different European countries with potential for differences in dietary intake, BMI, smoking status, and sociodemographic characteristics, the results of our study samples can mainly be generalized to women in high-income countries. However, it is worth noting that we found similar associations in EDEN, including mainly French pregnant women and Generation R, with a large proportion of children ethnicities other than Dutch (44%) (16). We focused on the family lifestyle patterns in the preconception and pregnancy period, however, family practices in the early childhood period influence the child development as well (4). Further research is warranted to investigate how the dynamic path of lifestyle changes throughout pregnancy until early childhood may have synergistic influences. Finally, a large difference in the prevalence of childhood obesity between advantaged and disadvantaged families is reported in the literature (12). We adjusted our analyses for some socioeconomic characteristics, however further research should be conducted to investigate the social determinants of the identified family lifestyle patterns, with a specific focus on socio-economic inequalities, migration history and geographic origin.

## 5. Conclusion

Among the various lifestyle patterns identified in the four European cohorts, the two explaining the most variance in pregnancy were characterized by “high parental smoking, low maternal diet quality or high maternal sedentary behavior” and, by “high parental BMI and low GWG.” The observed associations between the identified parental lifestyle patterns and the child’s risk of obesity from the age of 5 years underlines the importance of family and multi-behavioral approaches. Our findings represent a significant contribution to the knowledge regarding the importance of parental lifestyle factors from preconception. A better understanding of their social determinants will help increase the effectiveness of future childhood obesity prevention strategies in early life.

## Data availability statement

Datasets generated during and/or analysed during the current study are not publicly available but are available from the corresponding author on reasonable request.

## Ethics statement

The studies involving human participants were reviewed and approved by Ethics Committee of the Bicêtre Hospital for the EDEN study, Ethics committee (Comité de Protection des Personnes), the national committee on information concerning health research (Comité Consultatif sur le Traitement de l’Information en Matière de Recherche dans le domaine de la Santé), and the data protection authority (Commission Nationale de l’Informatique et des Libertés) for Elfe, the Medical Ethical Committee of the Erasmus Medical Center, Rotterdam for Generation R, the Research Ethics Committees in the Coombe University Hospital, Dublin, University College Dublin, Irish College of General Practitioners and University College Hospital, Galway, Ireland for the Lifeways study. The patients/participants provided their written informed consent to participate in this study.

## Author contributions

ML, RG, BH, and SL: conceptualization. ML, MG, MT, AD, JH, BL-G, and BH: data curation. ML, MS, AO’D, AA, BH, and SL: formal analysis. CP, RG, and BH: funding acquisition and resources. CP, RG, BH, and SL: investigation. ML, CP, RG, BH, and SL: methodology. CP, RG, CK, and BH: project administration. ML: software. BH and SL: supervision and validation. ML, BH, and SL: visualization and writing – original draft preparation. ML, MS, AO’D, AA, MT, MG, AD, JH, BL-G, CK, M-AC, CP, RG, BH, and SL: writing – review and editing. All authors contributed to the article and approved the submitted version.

## Funding

This project has received funding from the European Union’s Horizon 2020 research and innovation programme under the ERA-NET Cofund action (no. 727565), European Joint Programming Initiative “A Healthy Diet for a Healthy Life” (JPI HDHL, EndObesity). ML received a midwife research grant for this project from the Mustela Foundation. ML’s work has been also jointly supported by the French National College of Midwives and the Cerba Institute. The **EDEN** study was supported by Foundation for medical research (FRM), National Agency for Research (ANR), National Institute for Research in Public health (IRESP: TGIR cohorte santé 2008 program), French Ministry of Health (DGS), French Ministry of Research, INSERM Bone and Joint Diseases National Research (PRO-A), and Human Nutrition National Research Programs, Paris-Sud University, Nestlé, French National Institute for Population Health Surveillance (InVS), French National Institute for Health Education (INPES), the European Union FP7 programs (FP7/2007–2013, HELIX, ESCAPE, ENRIECO, Medall projects), Diabetes National Research Program (through a collaboration with the French Association of Diabetic Patients (AFD)), French Agency for Environmental Health Safety (now ANSES), Mutuelle Générale de l’Education Nationale a complementary health insurance (MGEN), French national agency for food security, and French-speaking association for the study of diabetes and metabolism (ALFEDIAM). The **Elfe** survey is a joint project between the French Institute for Demographic Studies (INED) and the National Institute of Health and Medical Research (INSERM), in partnership with the French blood transfusion service (Etablissement français du sang, EFS), Santé publique France, the National Institute for Statistics and Economic Studies (INSEE), the Direction générale de la santé (DGS, part of the Ministry of Health and Social Affairs), the Direction générale de la prévention des risques (DGPR, Ministry for the Environment), the Direction de la recherche, des études, de l’évaluation et des statistiques (DREES, Ministry of Health and Social Affairs), the Département des études, de la prospective et des statistiques (DEPS, Ministry of Culture), and the Caisse nationale des allocations familiales (CNAF), with the support of the Ministry of Higher Education and Research and the Institut national de la jeunesse et de l’éducation populaire (INJEP). Via the RECONAI platform, it receives a government grant managed by the National Research Agency under the “Investissements d'avenir” programme (ANR-11-EQPX-0038, ANR-19-COHO-0001). The general design of the **Generation R Study** is made possible by financial support from the Erasmus Medical Center, Rotterdam, Erasmus University Rotterdam, Netherlands Organization for Health Research and Development (ZonMw), Netherlands Organisation for Scientific Research, Ministry of Health, Welfare and Sport, and Ministry of Youth and Families. **The Lifeways cross-generation cohort** was supported by the Irish Health Research Board (ref JPI ERA NET HDHL INTIMIC 2020-1), grant provided to CMP; The funders have no role in the collection, analysis and interpretation of data; in the writing of the report; and in the decision to submit the article for publication.

## Conflict of interest

JH owns controlling interest in Connecting Health Innovations, a company that has licensed the right to his invention of the dietary inflammatory index from the University of South Carolina to develop computer and smart phone applications for patient counselling and dietary intervention in clinical settings.

The remaining authors declare that the research was conducted in the absence of any commercial or financial relationships that could be construed as a potential conflict of interest.

## Publisher’s note

All claims expressed in this article are solely those of the authors and do not necessarily represent those of their affiliated organizations, or those of the publisher, the editors and the reviewers. Any product that may be evaluated in this article, or claim that may be made by its manufacturer, is not guaranteed or endorsed by the publisher.

## Abbreviations

AR: adiposity rebound
BMI: body mass index
DASH: Dietary Approaches to Stop Hypertension
DII^®^: Dietary Inflammatory Index
E-DII™: energy-adjusted DII
FFQ: food-frequency questionnaire
GWG: gestational weight gain
OW-OB: overweight and obesity
PCA: principal component analyses
