# Supplementary material for: Impact of parental lifestyle patterns in the preconception and pregnancy periods on childhood obesity
Source: Front Nutr. 2023 May 18;10:1166981. doi: 10.3389/fnut.2023.1166981 (PMC10233059; doi:10.3389/fnut.2023.1166981)
Supplement: Supplementary file 1 [file Table_1.docx]

Supplementary Material

**Impact of parental lifestyle patterns in the preconception and pregnancy periods on childhood obesity**

**Marion Lecorguillé ^1^, Mireille C. Schipper ^2^, Aisling O’Donnell ^3^, Adrien M. Aubert ^3^, Muriel Tafflet ^1^, Malamine Gassama ^4^, Alexander Douglass ^3^, James R. Hébert ^5,6^, Blandine de Lauzon-Guillain ^1^, Cecily Kelleher ^3^, Marie-Aline Charles ^1,4^, Catherine M. Phillips ^3^, Romy Gaillard ^2^, Sandrine Lioret ^1,^**† **and Barbara Heude ^1,^**†

^1^Université Paris Cité and Université Sorbonne Paris Nord, Inserm, INRAE, Center for Research in Epidemiology and StatisticS (CRESS), Paris, France, ^2^ The Generation R Study Group (Na 29-15), Erasmus University Medical Center, CA, Rotterdam, Netherlands, ^3^ School of Public Health, Physiotherapy and Sports Science, University College Dublin, Dublin, Ireland, ^4^ Ined, Inserm, EFS, Joint Unit Elfe, Aubervilliers, France, ^5^ Cancer Prevention and Control Program and Department of Epidemiology and Biostatistics, Arnold School of Public Health, University of South Carolina, Columbia, SC, United States, ^6^ Department of Nutrition, Connecting Health Innovations, LLC, Columbia, SC, United States

†These authors have contributed equally to this work and share last authorship

*** Correspondence**: marion.lecorguille@inserm.fr

**Table S1 Information on data request contacts, full cohort recruitment date, and local institutional ethical review boards for each cohort.**

| **Cohort name (country)** | **Contact for data requests** | **Full cohort recruitment date (including day and month)** | **Local institutional ethical review boards** |
| --- | --- | --- | --- |
| EDEN (France) | [etude.eden@inserm.fr](mailto:etude.eden@inserm.fr) | 27 January 2003 to 6 March 2006 | Ethics Committee of the Bicêtre Hospital |
| Elfe (France) | <https://www.elfe-france.fr/>  [contact@elfe-france.fr](mailto:contact@elfe-france.fr) | Infants born between April and December 2011 | Ethics committee (Comité de Protection des Personnes), the national committee on information concerning health research (Comité Consultatif sur le Traitement de l’Information en Matière de Recherche dans le domaine de la Santé), and the data protection authority (Commission Nationale de l’Informatique et des Libertés) |
| Generation R (The Netherlands) | [generationr@erasmusmc.nl](mailto:generationr@erasmusmc.nl) | Pregnant women with an expected delivery date between April 2002 and January 2006 | Medical Ethical Committee of the Erasmus Medical Center, Rotterdam |
| Lifeways (Republic of Ireland) | [lifeways@ucd.ie](mailto:lifeways@ucd.ie) | 2 October 2001 to 4 April 2003 | Research Ethics Committees in the  Coombe University Hospital, Dublin, University College Dublin, Irish College of General Practitioners and University College Hospital, Galway, Ireland. |

**Table S2: List of candidate family-based lifestyle factors for patterns derivation for each period**

| **Variable** | **Description** | **Availability** |
| --- | --- | --- |
| **Pre-conception*** |  |  |
| Maternal smoking before pregnancy | No  <10 per day  ≥ 10 per day | All cohorts |
| Maternal pre-pregnancy weight | Continuous (If pre-pregnancy weight is not available, use early pregnancy weight closest to conception, limited to 1st trimester (<12 weeks). | All cohorts |
| Maternal height | Continuous | All cohorts |
| Maternal DASH/E-DII | Continuous  - DASH score with frequencies  - E-DII | EDEN, Gen R |
| Paternal smoking before pregnancy | None  <10 per day  ≥ 10 per day | EDEN, Gen R |
| Paternal BMI at inclusion | Continuous | EDEN, Elfe, Gen R |
| Paternal diet | Food groups consumption (meat/processed meat; candies chocolates/pastries/cakes/sweet beverages; chips/pizza/french fries; alcohol (beer, wine, other alcohols)) | Elfe |
| **Pregnancy period** |  |  |
| Maternal smoking | No  < 10 per day  ≥ 10 per day | All cohorts |
| Maternal pre-pregnancy BMI | Continuous (If pre-pregnancy weight is not available, use early pregnancy weight closest to conception, limited to 1st trimester (<12 weeks). | All cohorts |
| Maternal GWG | Continuous | Not in Lifeways |
| Maternal DASH/E-DII | Continuous | DASH : all cohorts  E-DII : in EDEN, Lifeways, Gen R |
| Maternal physical activity | -Eden: overall activity (occupational, sport, leisure-time activity) at first trimester  -Elfe: MET-hr/wk; household/caregiving, occupational settings, sports/exercise, sedentarity - the last 3 months  - Lifeways : METs equivalent at early pregnancy | Not in Gen R |
| Paternal smoking | 0 = No  1 = < 10 per day  2 = ≥ 10 per day | All cohorts, recoded as binary variable for Lifeways (non-smoker; smoker) |
| Paternal BMI at inclusion | Continuous | All cohorts |
| Paternal E-DII | Continuous | Lifeways only |
| Paternal physical activity | Continuous - METS-equivalent | Lifeways only |

## *Exclusion of Lifeways

**Table S3: Missing data and method used for imputation in the EDEN study**

| Variable | Type of variable | Model used to predict missing data* | Missing value  % |
| --- | --- | --- | --- |
| Centre | Binary | Logistic regression | 0.00 (0) |
| Household income | Categorical (4 categories) | Ordinal regression | 4.14 (82) |
| Cohabiting status | Binary | Logistic regression | 3.08 (61) |
| Marital status | Binary | Logistic regression | 3.38 (67) |
| Maternal age | Continuous | Linear regression | 3.74 (74) |
| Maternal place of birth | Binary | Logistic regression | 3.79 (75) |
| Maternal employment status | Binary | Logistic regression | 3.28 (65) |
| Maternal education | Categorical (3 categories) | Ordinal regression | 3.58 (71) |
| Universal complementary health insurance | Binary | Logistic regression | 3.38 (67) |
| Maternal pre-pregnancy BMI | Continuous | Linear regression | 4.90 (97) |
| Maternal pre-pregnancy smoking | Categorical (3 categories) | Ordinal regression | 4.04 (80) |
| Maternal GWG | Continuous | Linear regression | 5.75 (114) |
| Maternal pregnancy smoking | Categorical (3 categories) | Ordinal regression | 17.82 (353) |
| Maternal pregnancy E-DII | Continuous | Linear regression | 19.28 (382) |
| Maternal pregnancy DASH | Continuous | Linear regression | 6.66 (132) |
| Maternal pre-pregnancy E-DII | Continuous | Linear regression | 14.0 (273) |
| Maternal pre-pregnancy DASH | Continuous | Linear regression | 0.8 (17) |
| Maternal Sport PA | Binary | Logistic regression | 3.89 (77) |
| Maternal leisure PA | Continuous | Linear regression | 2.93 (58) |
| Maternal work PA | Continuous | Linear regression | 4.29 (85) |
| Parity | Binary | Logistic regression | 3.94 (78) |
| Paternal age | Continuous | Linear regression | 13.23 (262) |
| Paternal place of birth | Binary | Logistic regression | 3.94 (78) |
| Paternal employment status | Binary | Logistic regression | 5.35 (106) |
| Paternal education | Categorical (3 categories) | Ordinal regression | 3.53 (70) |
| Paternal pre-pregnancy smoking | Categorical (3 categories) | Ordinal regression | 14.84 (294) |
| Paternal BMI | Continuous | Linear regression | 9.99 (198) |
| Paternal pregnancy smoking | Categorical (3 categories) | Ordinal regression | 12.62 (250) |
| Breastfeeding | Continuous | Linear regression | 4.543 (90) |
| Child BMI z score at 5 years* | Continuous | Linear regression | 42.30 (838) |
| Child BMI z score at 8 years* | Continuous | Linear regression | 62.80 (1244) |
| Child BMI z score at 12 years* | Continuous | Linear regression | 64.36 (1275) |
| Age at adiposity rebound* | Continuous | Linear regression | 28.57 (566) |
| BMI at adiposity rebound* | Continuous | Linear regression | 28.57 (566) |
| Age at adiposity peak | Continuous | Linear regression | 13.53 (268) |
| BMI at adiposity peak | Continuous | Linear regression | 13.53 (268) |
| Child gender | Binary | Logistic regression | 3.9 (78) |

*Outcomes were used for the prediction, but observed values (and not imputed values) were used for further analyses.

**Table S4: Missing data and method used for imputation in the Elfe study**

| Variable | Type of variable | Model used to predict missing data* | Missing value  % |
| --- | --- | --- | --- |
| Household income | Categorical (4 categories) | Ordinal regression | 14.69 (2630) |
| Cohabiting status | Binary | Logistic regression | 0.84 (151) |
| Maternal age | Continuous | Linear regression | 0.38 (68) |
| Maternal place of birth | Binary | Logistic regression | 5.38 (963) |
| Maternal employment status | Binary | Logistic regression | 0.55 (99) |
| Maternal education | Categorical (3 categories) | Ordinal regression | 0.02 (4) |
| Pregnancy follow-up | Categorical (3 categories) | Multinomial regression | 1.2 (214) |
| Health insurance | Binary | Logistic regression | 0.82 (147) |
| Maternal pre-pregnancy BMI | Continuous | Linear regression | 1.597 (286) |
| Maternal pre-pregnancy smoking | Categorical (3 categories) | Ordinal regression | 1.64 (293) |
| Maternal GWG | Continuous | Linear regression | 2.30 (411) |
| Maternal pregnancy smoking | Categorical (3 categories) | Ordinal regression | 1.37 (246) |
| Maternal pregnancy DASH | Continuous | Linear regression | 14.97 (2681) |
| Maternal Sport PA | Continuous | Linear regression | 15.16 (2714) |
| Maternal occupation PA | Continuous | Linear regression | 14.70 (2631) |
| Maternal Household PA | Continuous | Linear regression | 14.18 (2538) |
| Maternal sedentary PA | Continuous | Linear regression | 14.44 (2586) |
| Parity | Binary | Logistic regression | 1.34 (239) |
| Paternal age | Continuous | Linear regression | 4.66 (835) |
| Paternal place of birth | Binary | Logistic regression | 8.31 (1487) |
| Paternal employment status | Binary | Logistic regression | 2.26 (405) |
| Paternal education | Categorical (3 categories) | Ordinal regression | 23.69 (4241) |
| Paternal BMI | Continuous | Linear regression | 30.45 (5451) |
| Paternal pregnancy smoking | Categorical (3 categories) | Ordinal regression | 30.21 (5409) |
| Paternal walk PA per day | Binary | Logistic regression | 94.68 (16952) |
| Paternal PA per week | Binary | Logistic regression | 94.40 (16902) |
| Paternal meat consumption | Continuous | Linear regression | 94.43 (16906) |
| Paternal sugar foods consumption | Continuous | Linear regression | 94.43 (16906) |
| Paternal salt and fat foods consumption | Continuous | Linear regression | 94.43 (16906) |
| Paternal meat consumption | Continuous | Linear regression | 94.43 (16906) |
| Paternal fruits and vegetables consumption | Continuous | Linear regression | 94.43 (16906) |
| Paternal nuts consumption | Continuous | Linear regression | 94.43 (16906) |
| Paternal whole grains consumption | Continuous | Linear regression | 94.43 (16906) |
| Breastfeeding | Continuous | Linear regression | 15.68 (2808) |
| Child BMI z score at 5 years* | Continuous | Linear regression | 47.86 (8569) |
| Child BMI z score at 8 years* | Continuous | Linear regression | 78.63 (14078) |
| Child BMI z score at 12 years* | Continuous | Linear regression | 81.35 (14565) |
| Age at adiposity rebound* | Continuous | Linear regression | 56.62 (10137) |
| BMI at adiposity rebound* | Continuous | Linear regression | 56.62 (10137) |
| Age at adiposity peak | Continuous | Linear regression | 35.97 (6441) |
| BMI at adiposity peak | Continuous | Linear regression | 35.97 (6441) |
| Child Gender | Binary | Logistic regression | 0.06 (12) |

*Outcomes were used for the prediction, but observed values (and not imputed) were used for further analyses.

**Table S5: Missing data and method used for imputation in the Generation R study**

| Variable | Type of variable | Model used to predict missing data* | Missing value  % |
| --- | --- | --- | --- |
| Household income | Categorical (3 categories) | Ordinal regression | 25.96 (2275) |
| Maternal age | Continuous | Linear regression | 0.03 (3) |
| Maternal place of birth | Binary | Logistic regression | 3.5 (307) |
| Maternal employment status | Binary | Logistic regression | 25.31 (2218) |
| Maternal education | Categorical (3 categories) | Ordinal regression | 6.88 (603) |
| Maternal pre-pregnancy BMI | Continuous | Linear regression | 17.82 (1562) |
| Maternal pre-pregnancy smoking | Categorical (3 categories) | Ordinal regression | 17.7 (1551) |
| Maternal GWG | Continuous | Linear regression | 21.69 (1901) |
| Maternal pregnancy smoking | Categorical (3 categories) | Ordinal regression | 18.03 (1580) |
| Maternal pregnancy E-DII | Continuous | Linear regression | 28.68 (2514) |
| Maternal pregnancy DASH | Continuous | Linear regression | 28.68 (2514) |
| Parity | Categorical (3 categories (0, 1 and 2) | Multinomial regression | 2.07 (181) |
| Paternal age | Continuous | Linear regression | 17.74 (1555) |
| Paternal place of birth | Binary | Logistic regression | 10.8 (947) |
| Paternal employment status | Binary | Logistic regression | 45.57 (3994) |
| Paternal education | Categorical (3 categories) | Ordinal regression | 41.36 (3625) |
| Paternal BMI | Continuous | Linear regression | 29.52 (2587) |
| Paternal pregnancy smoking | Categorical (3 categories) | Ordinal regression | 16.41 (1438) |
| Breastfeeding | Continuous | Linear regression | 44.46 (3897) |
| Child BMI z score at 5 years* | Continuous | Linear regression | 30.21 (2648) |
| Child BMI z score at 9 years* | Continuous | Linear regression | 40.37 (3538) |
| Child gender | Binary | Logistic regression | 0.05 (4) |
| Age at adiposity rebound* | Continuous | Linear regression | 28.13 (2466) |
| BMI at adiposity rebound* | Continuous | Linear regression | 28.13 (2466) |
| Age at adiposity peak | Continuous | Linear regression | 30.06 (2635) |
| BMI at adiposity peak | Continuous | Linear regression | 30.06 (2635) |

*Outcomes were used for the prediction, but observed values (and not imputed) were used for further analyses.

**Table S6: Missing data and method used for imputation in the Lifeways study**

| Variable | Type of variable | Model used to predict missing data* | Missing value  % |
| --- | --- | --- | --- |
| Household income | Binary | Logistic regression | 9.44 (88) |
| Maternal age | Continuous | Linear regression | 0.64 (6) |
| Maternal employment status | Binary | Logistic regression | 0.97 (9) |
| Maternal education | Categorical (3 categories) | Ordinal regression | 2.79 (26) |
| Maternal pre-pregnancy BMI | Continuous | Linear regression | 17.70 (165) |
| Maternal pre-pregnancy smoking | Binary | Logistic regression | 17.17 (160) |
| Maternal pregnancy smoking | Categorical (3 categories) | Ordinal regression | 4.51 (42) |
| Maternal pregnancy E-DII | Continuous | Linear regression | 0.43 (4) |
| Maternal pregnancy DASH | Continuous | Linear regression | 0.43 (4) |
| Maternal Sport PA | Binary | Logistic regression | 18.24 (170) |
| Parity | Binary | Logistic regression | 1.50 (14) |
| Paternal age | Continuous | Linear regression | 13.73 (128) |
| Paternal employment status | Binary | Logistic regression | 18.67 (174) |
| Paternal education | Categorical (3 categories) | Ordinal regression | 9.87 (92) |
| Paternal BMI | Continuous | Linear regression | 71.35 (665) |
| Paternal pre-pregnancy smoking | Binary | Logistic regression | 73.50 (685) |
| Paternal pregnancy smoking | Binary | Logistic regression | 71.14 (663) |
| Paternal E-DII | Continuous | Linear regression | 68.03 (634) |
| Paternal Sport PA | Continuous | Linear regression | 70.60 (658) |
| Child BMI z score at 5 years* | Continuous | Linear regression | 42.70 (398) |
| Child BMI z score at 10 years* | Continuous | Linear regression | 69.00 (643) |

*Outcomes were used for the prediction, but observed values (and not imputed) were used for further analyses.

**Table S7: Principal component analysis factor loadings for the family lifestyle patterns in the preconception and pregnancy periods**

| **Variables** | **Lifestyle pattern 1** | **Lifestyle pattern 2** | **Lifestyle pattern 3** |
| --- | --- | --- | --- |
| **EDEN study** |  |  |  |
| **Preconception period (N=1981)** |  |  |  |
| Maternal smoking before pregnancy | 0.64* | -0.24 | 0.50* |
| Maternal pre-pregnancy BMI | -0.07 | 0.66* | 0.42* |
| Maternal E-DII | 0.75* | 0.27 | -0.42* |
| Maternal DASH | -0.77* | -0.29 | 0.36* |
| Paternal BMI at inclusion | -0.10 | 0.71* | 0.26 |
| Paternal smoking before pregnancy | 0.62* | -0.25 | 0.52* |
| Percentage of explained variance^a^ | 32.9 | 20.4 | 17.8 |
| **Pregnancy period (N=1962)** |  |  |  |
| Maternal smoking before pregnancy | 0.51* | 0.14 | 0.45* |
| Maternal pre-pregnancy BMI | -0.04 | -0.74* | 0.3 |
| Maternal GWG | 0.17 | 0.51* | -0.41* |
| Maternal E-DII | 0.76* | 0.002 | -0.24 |
| Maternal DASH | -0.78* | 0.09 | 0.15 |
| Maternal Sport PA | -0.20 | 0.29 | 0.27 |
| Maternal occupational PA | -0.13 | 0.04 | -0.44* |
| Maternal leisure PA | -0.48* | 0.34* | 0.19 |
| Paternal BMI at inclusion | -0.05 | -0.54* | -0.29 |
| Paternal smoking | 0.5* | 0.17 | 0.45* |
| Percentage of explained variance^a^ | 20.2 | 13.6 | 11.3 |
| **Elfe study** |  |  |  |
| **Preconception period (N=917)** |  |  |  |
| Maternal pre-pregnancy BMI | 0.06 | 0.69* | 0.23 |
| Maternal pre-pregnancy smoking | 0.02 | 0.37* | -0.55* |
| Paternal BMI at inclusion | 0.02 | 0.64* | 0.49* |
| Paternal meat consumption | 0.65* | 0.04 | 0.23 |
| Paternal fruits/vegetables consumption | -0.06 | -0.45* | 0.62* |
| Paternal sugar | 0.7* | -0.18 | -0.02 |
| Paternal salt/fast food | 0.75* | 0.01 | -0.15 |
| Percentage of explained variance^a^ | 0.21 | 0.18 | 0.15 |
| **Pregnancy period (N=17904)** |  |  |  |
| Maternal pre-pregnancy BMI | 0.25 | -0.74* | -0.1 |
| Maternal smoking | 0.66* | 0.25 | -0.03 |
| Maternal GWG | -0.02 | 0.68* | -0.02 |
| Maternal DASH | -0.63* | -0.05 | 0.17 |
| Maternal Sport PA | -0.08 | 0.02 | 0.77* |
| Maternal occupation PA | 0.01 | 0.02 | 0.54* |
| Maternal household PA | 0.3* | -0.2 | 0.57* |
| Maternal sedentary behavior | 0.41* | -0.05 | 0.14 |
| Paternal BMI at inclusion | 0.16 | -0.42* | -0.13 |
| Paternal smoking | 0.67* | 0.22 | 0.00 |
| Percentage of explained variance^a^ | 16.3 | 13.4 | 12.8 |
| **Generation R** |  |  |  |
| **Preconception period (N=8352)** |  |  |  |
| Maternal pre-pregnancy BMI | 0.12 | 0.73* | 0.16 |
| Maternal pre-pregnancy smoking | 0.6* | -0.3* | 0.49* |
| Maternal E-DII | 0.76* | 0.08 | -0.43* |
| Maternal DASH | -0.76* | -0.17 | 0.41* |
| Paternal BMI at inclusion | 0.07 | 0.68* | 0.37* |
| Paternal smoking | 0.6* | -0.24 | 0.51* |
| Percentage of explained variance^a^ | 31.8 | 19.7 | 16.9 |
| **Pregnancy period (N=8765)** |  |  |  |
| Maternal pre-pregnancy BMI | 0.1 | -0.73* | NA |
| Maternal pregnancy smoking | 0.62* | 0.26 | NA |
| Maternal E-DII | 0.76* | -0.13 | NA |
| Maternal DASH | -0.76* | 0.21 | NA |
| Maternal GWG | 0.1 | 0.68* | NA |
| Paternal BMI | 0.05 | -0.39* | NA |
| Paternal smoking | 0.59* | 0.2 | NA |
| Percentage of explained variance^a^ | 27.2 | 18.9 | NA |
| **Lifeways** |  |  |  |
| **Pregnancy period (N=932)** |  |  |  |
| Maternal pregnancy BMI | 0.00 | NA | NA |
| Maternal pregnancy smoking | 0.45* | NA | NA |
| Maternal E-DII | 0.80* | NA | NA |
| Maternal DASH | -0.80* | NA | NA |
| Maternal PA | -0.15 | NA | NA |
| Paternal BMI at inclusion | -0.11 | NA | NA |
| Paternal smoking | 0.50* | NA | NA |
| Paternal E-DII | 0.65* | NA | NA |
| Paternal PA | -0.28 | NA | NA |
| Percentage of Explained variation^a^ | 25.2 | NA | NA |

^a^Explained variation in all lifestyle factors from the PCA. * Factor loading ≥ 0.30.

**Table S8: Associations of preconception family lifestyle patterns with child BMI z-score between 5 and 12 years**

| **Child BMI z-score**  Adjusted  β [95% CI] | | | | | | | | | | |
| --- | --- | --- | --- | --- | --- | --- | --- | --- | --- | --- |
| **EDEN** | | | | **Elfe** | | | | **Gen R** | | |
|  | **5.5 years (N=1143)** | **8 years**  **(N=737)** | **12 years (N=706)** |  | **5 years (N=638)** | **7 years (N=493)** | **9 years (N=352)** |  | **5 years (N=5778)** | **9 years (N=4944)** |
| “Parental smoking and low maternal diet quality  ” | 0.00  [-0.04;0.04] | -0.05  [-0.10;0.01] | 0.02  [-0.04;0.07] | “Low paternal diet quality” | -0.06  [-0.14;0.02] | -0.03  [-0.12;0.06] | -0.12  [-0.25;0.00] | “High parental smoking, and low maternal diet quality” | 0.04  [0.02, 0.06]* | 0.05  [0.03, 0.07]* |
| “High parental BMI and low smoking” | 0.18  [0.14;0.23]* | 0.20  [0.14;0.27]* | 0.24  [0.18;0.31]* | “High parental BMI, maternal smoking and low paternal consumption of vegetables” | 0.22 [0.14;0.30]* | 0.21 [0.12;0.30]* | 0.36 [0.24;0.49]* | “High parental BMI and low smoking” | 0.22  [0.19, 0.24]* | 0.27  [0.25, 0.29]* |
| “High parental smoking, BMI and high maternal diet quality” | 0.14  [0.09;0.19]* | 0.15  [0.09;0.22]* | 0.2  [0.13;0.28]* | “High paternal BMI, high consumption of vegetables and low maternal smoking” | 0.18 [0.10;0.26]* | 0.11 [0.02;0.19]* | 0.18 [0.07;0.30]* | “High parental smoking, high paternal BMI, and high maternal diet quality” | 0.15  [0.12, 0.17]* | 0.16  [0.13, 0.18]* |

Note: Lifeways study is not included because of the lack of information on behaviors during the pre-conception period.

Models are adjusted for parental age, born abroad or not, parental education, parental employment status, parity, household income.

Results are presented using imputed lifestyle patterns (except for Elfe). *significant associations p <0.05. β values of linear regression and 95% CIs for 1 SD increase in lifestyle pattern score.

**Table S9: Associations of preconception maternal lifestyle patterns with child BMI z-score, risk of overweight and age at adiposity rebound between 5 and 12 years**

| **Child BMI z-score**  Adjusted  β [95% CI] | | | | | | | | |
| --- | --- | --- | --- | --- | --- | --- | --- | --- |
| **EDEN** | | | | **Gen R** | | | | |
|  | **5.5 years (N=1143)** | **8 years (N=737)** | **12 years (N=706)** |  | | **5 years (N=5686)** | | **9 years (N=4855)** |
| “High smoking and dietary  inflammatory potential & low DASH” | 0.01  [-0.03;0.05] | -0.03  [-0.09;0.02] | 0.01  [-0.05;0.07] | “High smoking,  dietary inflammatory potential  & low DASH” | | 0.02  [0.00; 0.04]* | | 0.04  [0.02 ; 0.06]* |
| “High BMI and rather low smoking” | 0.16  [0.11;0.21]* | 0.18  [0.11;0.26]* | 0.21  [0.13;0.29]* | “High BMI and low smoking” | | 0.20  [0.17; 0.22]* | | 0.24  [0.21; 0.27]* |
| **IOTF overweight/obesity****  Adjusted  OR [95% CI] | | | | | | | | |
| **EDEN** | | | | **Gen R** | | | | |
|  | **5.5 years (N=1143)** | **8 years (N=737)** | **12 years (N=706)** |  | | **5 years (N=5686)** | | **9 years (N=4855)** |
| “High smoking and dietary  inflammatory potential & low DASH” | 1.15  [0.95-1.40] | 0.93  [0.76-1.13] | 1.21  [0.99-1.48] | “High smoking, dietary inflammatory potential & low DASH” | | 1.09  [1.03- 1.16]* | | 1.13  [1.06-1.21]* |
| “High BMI and rather low smoking” | 1.53  [1.27-1.85]* | 1.61  [1.29-2.01]* | 1.41  [1.12-1.78]* | “High BMI and low smoking” | | 1.52  [1.42-1.63]* | | 1.58  [1.46-1.71]* |
| **Age at adiposity rebound (days)**  Adjusted  β [95% CI] | | | | | | | | |
| **EDEN (N=1415)** | | | | **Gen R (N=5833)** | | | | |
| “High smoking and dietary  inflammatory potential & low DASH” | | -12.7  [-34.1 ; 8.8] | | | “High smoking, dietary inflammatory potential & low DASH” | | -13.9  [-24.0; -3.8]* | |
| “High BMI and rather low smoking” | | -78.6  [-105.4; -51.9]* | | | “High BMI and low smoking” | | -96.9  [-109.7; -84.0]* | |

Elfe and Lifeways were not included because of the lack of information on behaviors during the pre-conception period.

Note: Models are adjusted for parental age, born abroad or not, parental education, parental employment status, parity, household income. When the outcome is the age of AR, models were further adjusted for child sex.

Results are presented using imputed lifestyle patterns. *significant associations p <0.05. **IOTF overweight and obesity vs underweight and normal BMI (reference). β values and OR values of linear and logistic regression and 95% CIs for 1 SD increase in lifestyle pattern score.

**Table S10: Associations of pregnancy family lifestyle patterns with child BMI z-score between 5 and 12 years**

|  |  |  |  | |  |  |  | |  | | **Child BMI z-score**  Adjusted  β [95% CI] | | | |  | | | |  |  | |  | | |  | |  | |
| --- | --- | --- | --- | --- | --- | --- | --- | --- | --- | --- | --- | --- | --- | --- | --- | --- | --- | --- | --- | --- | --- | --- | --- | --- | --- | --- | --- | --- |
| **EDEN** | | | | | | | | **Elfe** | | | | | | | | **Gen R** | | | | | | | **Lifeways** | | | | | |
|  | **5.5 years (N=1143)** | | | **8 years (N=737)** | | | **12 years (N=706)** | | |  | **5 years (N=9335)** | **7 years (N=3826)** | **9 years (N=3339)** | | | |  | | **5 years (N=6117)** | | **9 years (N=5227)** | | |  | | **5 years (N=534)** | | **9 years (N=289)** |
| “High parental smoking, low maternal diet quality and low leisure PA” | 0.01  [-0.03;0.05] | | | 0.00  [-0.05;0.06] | | | 0.02  [-0.04;0.08] | | | “High parental smoking, low maternal diet quality and maternal sedentary” | 0.09 [0.07;0.11]* | 0.11 [0.08;0.14]* | 0.10 [0.07;0.13]* | | | | “High parental smoking, low maternal diet quality” | | 0.04  [0.02, 0.06]* | | 0.05  [0.03, 0.07]* | | | “High parental smoking, inflammatory diet, low maternal DASH and low paternal PA” | | -0.02  [-0.08;0.04] | | 0.05 [-0.04;0.14] |
| “Low parental BMI and high GWG” | -0.13  [-0.18;-0.09]* | | | -0.17  [-0.23;-0.10]* | | | -0.19  [-0.25;-0.12]* | | | “Low parental BMI and high GWG” | -0.11  [-0.13; -0.09]* | -0.15  [-0.18;-0.13]* | | -0.18  [-.0.21;-0.15]* | | | | “Low parental BMI, high GWG” | -0.12  [-0.14, -0.09]* | | -0.15  [-0.18, -0.13]* | | |  | |  | |  |
| “Parental smoking, low GWG and low maternal work PA” | 0.01  [-0.04;0.06] | | | -0.03  [-0.10;0.04] | | | 0.03  [-0.05;0.10] | | | “High maternal physical activity” | -0.02  [-0.03;0.00] | 0.00  [-0.03;0.03] | -0.04  [-0.07;0.00] | | | |  | |  | |  | | |  | |  | |  |
|  |  | | |  | | |  | | | “High GWG, sedentary and occupation PA” | 0.07 [0.05;0.09]* | 0.09 [0.06;0.13]* | 0.06 [0.03;0.10]* | | | |  | |  | |  | | |  | |  | |  |

Note: Models are adjusted for parental age, born abroad or not, parental education, parental employment status, parity, household income.

For Lifeways, models were not adjusted for paternal employment status (almost all cases reported being employed/self-employed) and on country of birth (maternal birth outside Ireland was an exclusion criterion).

*significant associations p <0.05. β values of linear regression and 95% CIs for 1 SD increase in lifestyle pattern score.

**Table S11: Associations between pregnancy maternal lifestyle patterns and child BMI z-score, risk of overweight between 5 and 12 years and age at adiposity rebound**

| **Child BMI z-score**  Adjusted  β [95% CI] | | | | | | | | | | | | | | | | | | | | | |
| --- | --- | --- | --- | --- | --- | --- | --- | --- | --- | --- | --- | --- | --- | --- | --- | --- | --- | --- | --- | --- | --- |
| **EDEN** | | | | | | **Elfe** | | | | | | **Gen R** | | | | | **Lifeways** | | | | |
|  | **5.5 years (N=1143)** | | **8 years (N=737)** | **12 years (N=706)** | |  | | **5 years (N=9324)** | **7 years (N=3821)** | | **9 years (N=3334)** |  | | **5 years (N=6019)** | **9 years (N=5149)** | |  | **5 years (N=534)** | | | **9 years (N=289)** |
| “Low smoking, high diet quality and leisure PA” | -0.02  [-0.06;0.02] | | -0.02  [-0.08;0.03] | -0.02  [-0.08;0.04] | | “High BMI, smoking, low diet quality, high household PA, and sedentary” | | 0.11 [0.08;0.13]* | 0.14 [0.11;0.17]* | | 0.13 [0.10;0.17]* | “High BMI, smoking, low diet  quality” | | 0.03 [0.01, 0.05]* | 0.05 [0.05, 0.07]* | | “Smoking, and low diet quality” | | -0.02  [-0.09; 0.05] | | 0.07  [-0.04;0.18] |
| “Low BMI and high GWG” | -0.08  [-0.12;-0.03]* | | -0.09  [-0.15;-0.02]* | -0.11  [-0.19;-0.04]* | | “High diet quality, and high PA” | | -0.02  [-0.04;0.00] | 0;01  [-0.02;0.03] | | -0.01  [-0.05;0.02] | “Low BMI and high GWG, and smoking” | | -0.05 [-0.07, -0.03]* | -0.07 [-0.10, -0.05]* | | “Low maternal BMI & High maternal PA” | | -0.10  [-0.18; -0.01]* | | -0.11 [-0.25;0.02] |
| “Smoking and high sport PA” | 0.02  [-0.03;0.07] | | -0.01  [-0.07;0.06] | 0.03  [-0.04;0.10**]** | | “Low BMI, high GWG, smoking and high sport PA” | | -0.03  [-0.05;-0.01]* | -0.05  [-0.08;-0.02]* | | -0.10  [-0.14;-0.07]* |  | |  |  | |  | |  | |  |
| **IOTF overweight/obesity****  Adjusted  OR [95% CI] | | | | | | | | | | | | | | | | | | | | | |
| **EDEN** | | | | | | **Elfe** | | | | | | **Gen R** | | | | | **Lifeways** | | | | |
|  | **5.5 years (N=1143)** | | **8 years (N=737)** | **12 years (N=706)** | |  | | **5 years (N=9324)** | **7 years (N=3821)** | | **9 years (N=3334)** |  | | **5 years (N=6019)** | **9 years (N=5149)** | |  | **5 years (N=534)** | | | **9 years (N=289)** |
| “Low smoking, high diet quality and leisure PA” | 0.86 [0.72-1.03] | | 0.86 [0.71-1.05] | 0.91 [0.75-1.10] | | “High BMI, smoking, low diet quality, high household PA, and sedentary” | | 1.31 [1.23-1.40]* | 1.43 [1.29-1.58]* | | 1.37 [1.23-1.52]* | “High BMI, smoking, low diet quality” | | 1.12 [1.05- 1.18]* | 1.15 [1.08- 1.22]* | | “Smoking, and low diet quality” | 1.00 [0.84-1.19] | | | 1.11 [0.87-1.43] |
| “Low BMI and high GWG” | 0.81 [0.68-0.98]* | | 0.87 [0.70-1.07] | 0.87 [0.70-1.09] | | “High diet quality, and high PA” | | 0.91 [0.85-0.98]* | 1.00 [0.90-1.11] | | 0.95 [0.85-1.06] | “Low BMI and high GWG, and smoking” | | 0.87 [0.82- 0.92]* | 0.83 [0.78- 0.89]* | | “Low maternal BMI & High maternal PA” | 0.86 [0.70-1.04] | | | 0.75 [0.54-1.04] |
| “Smoking and high sport PA” | 1.15 [0.92-1.42] | | 0.95 [0.74-1.21] | 1.28 [1.03-1.59]* | | “Low BMI, high GWG, smoking and high sport PA” | | 0.98 [0.91-1.04] | 0.89 [0.80-0.98]* | | 0.84 [0.76-0.94]* |  | |  |  | |  |  | | |  |
| **Age at adiposity rebound (days)**  Adjusted  β [95% CI] | | | | | | | | | | | | | | | | | | | | | |
| **EDEN (N=1415)** | | | | | | **Elfe (N=7759)** | | | | |  | **Gen R (N=6198)** | | | | |  | **Lifeways** | | |  |
| “Low smoking, high diet quality and leisure PA” | | 15.6 [-5.5; 36.6] | | | “High BMI, smoking, low diet quality, high household PA, and sedentary” | | -47.4 [-56.3; -38.6]* | | | “High BMI, smoking, low diet quality” | | | -18.9 [-28.8, -8.9]* | | |  | | | | NA | |
| “Low BMI and high GWG” | | 45.0 [20.4; 69.6]* | | | “High diet quality, and high PA” | | 7.8 [-0.5; 16.0] | | | “Low BMI and high GWG, and smoking” | | | 29.9 [18.6; 41.1]* | | |  | | | |  | |
| “Smoking and high sport PA” | | 8.6 [-16.7; 33.9] | | | “Low BMI, high GWG, smoking and high sport PA” | | 21.1 [12.3; 30.0]* | | |  | | |  | | |  | | | |  | |

Note: Models are adjusted for parental age, born abroad or not, parental education, parental employment status, parity, household income. When the outcome is the age of AR, models were further adjusted for child sex.

For Lifeways, models were not adjusted for paternal employment status (almost all cases reported being employed/self-employed) and on country of birth (maternal birth outside Ireland was an exclusion criterion).

Results are presented using imputed lifestyle patterns. *significant associations p <0.05. **IOTF overweight and obesity vs underweight and normal BMI (reference). β values and OR values of linear and logistic regression and 95% CIs for 1 SD increase in lifestyle pattern score.

**Supplementary Text 1**

**Dietary scores**

The DASH diet is widely promoted in particular for the prevention and treatment of hypertension (1). The DASH score in ALPHABET was generated based mainly on the index proposed by Fung and colleagues (1), which aims to classify women into quintiles according to their intake ranking. The DASH score was composed of 8 food components. A high score corresponds to high intakes of total grains, vegetables (excluding potatoes and condiments), fruits, non-full-fat dairy products, and nuts/seeds/legumes, and low intakes of red and processed meats, sugar-sweetened beverages/sweets/added sugars, and sodium). We used the DASH score with frequencies (not intrinsically adjusted for energy intake). Dietary inflammatory potential was determined using the energy-adjusted DII (E-DII), a validated literature-derived score from the original DII, calculation of which has been previously described in detail (2). The DII combines a range of macronutrients and micronutrients which either increased or decreased circulating biomarkers of inflammation (CRP, IL1-β, IL-4, IL-6, IL-10, and TNFα) (2) and was designed to be universally applicable across all human studies with adequate dietary assessment (3). However, the observation that the relationship between DII and energy intake is complex and differs across data led to the development of the E-DII (3). A higher E-DII score indicates a more pro-inflammatory diet. The E-DII score in ALPHABET was generated from 25 to 28 (out of 44 possible) dietary parameters in all cohorts (except for Generation R, which has 20 dietary parameters) (4). Dietary parameters include beta-carotene, carbohydrate, cholesterol, alcohol, fiber, protein, fat, saturated fat, mono-unsaturated fat, poly-unsaturated fat, trans-fat, omega 3, omega 6, niacin, thiamin, riboflavin, vitamin A, vitamin C, vitamin B12, vitamin B6, vitamin D, vitamin E, folic acid, iron, magnesium, zinc, selenium, onion, garlic, tea and caffeine.

**Physical activity**

The Baecke questionnaire includes a total of 16 questions scored on a five-point Likert scale and classified into three domains: Occupational, Sports, and Leisure-time activity (average of four usual leisure activities: watching TV, walking, biking, swimming; and the number of minutes spent per day on locomotion activities (walking or riding a bike to and from work or shops). Scoring for the three domains follows the system described by Baecke et al (5) with scores ranging from 1 (low) to 5 (high).

The Pregnancy Physical Activity Questionnaire included the time spent in household/caregiving, occupational, sports/exercise, and sedentary activities during the last three months of pregnancy (6). This last domain includes questions on watching TV or a video, sitting and reading, talking or on the phone, and time spent driving or being in public transports. Self-reported time spent in each activity was multiplied by its intensity (pregnancy-specific estimates for walking and light- to moderate intensity household tasks, and Ainsworth compendium-based Metabolic Equivalent of Task (MET) values (7) for the remainder of the PPAQ activities to obtain a measure of average weekly energy expenditure (MET-hr/wk) attributable to each activity and domain. Scores have been calculated from all available data.

In Lifeways, mothers and fathers were asked “considering a 7-day period (a week), how many times on average do you do the following kinds of exercise (mild, moderate and strenuous) for more than 20 minutes during your free/leisure time?”. Strenuous exercise included notably running, jogging, vigorous swimming, and long-distance cycling); moderate exercise included fast walking, tennis, easy swimming, cycling, heavy gardening; while mild exercise included yoga, golf, easy walking, light gardening).

1. Fung TT, Chiuve SE, McCullough ML, Rexrode KM, Logroscino G, Hu FB. Adherence to a DASH-style diet and risk of coronary heart disease and stroke in women. Arch Intern Med. 2008;168(7):713-20.

2. Shivappa N, Steck SE, Hurley TG, Hussey JR, Hébert JR. Designing and developing a literature-derived, population-based dietary inflammatory index. Public Health Nutr. 2014;17(8):1689-96.

3. Hébert JR, Shivappa N, Wirth MD, Hussey JR, Hurley TG. Perspective: The Dietary Inflammatory Index (DII)-Lessons Learned, Improvements Made, and Future Directions. Adv Nutr. 2019;10(2):185-95.

4. Chen LW, Aubert AM, Shivappa N, Bernard JY, Mensink-Bout SM, Geraghty AA, et al. Associations of maternal dietary inflammatory potential and quality with offspring birth outcomes: An individual participant data pooled analysis of 7 European cohorts in the ALPHABET consortium. PLoS Med. 2021;18(1):e1003491.

5. Baecke JA, Burema J, Frijters JE. A short questionnaire for the measurement of habitual physical activity in epidemiological studies. Am J Clin Nutr. 1982;36(5):936-42.

6. van der Waerden J, Nakamura A, Pryor L, Charles MA, El-Khoury F, Dargent-Molina P. Domain-specific physical activity and sedentary behavior during pregnancy and postpartum depression risk in the French EDEN and ELFE cohorts. Prev Med. 2019;121:33-9.

7. Ainsworth BE, Haskell WL, Herrmann SD, Meckes N, Bassett DR, Jr., Tudor-Locke C, et al. 2011 Compendium of Physical Activities: a second update of codes and MET values. Med Sci Sports Exerc. 2011;43(8):1575-81.
